# Acute Kidney Injury in Internal Medicine Wards During a Period of Wartime Healthcare System Disruption: A Retrospective Cohort Study

**DOI:** 10.3390/jcm15082943

**Published:** 2026-04-13

**Authors:** Nomy Levin Iaina, Naal Al’Turi, Yehonatan Marziano

**Affiliations:** 1 Department of Nephrology and Hypertension, Barzilai University Medical Center, Ashkelon 7830604, Israel; 2 School of Medicine, Faculty of Health Sciences, Ben Gurion University of the Negev, Beer Sheva 8410501, Israel; 3 Department of Medicine, Barzilai University Medical Center, Ashkelon 7830604, Israel; altorynael@gmail.com (N.A.); yehonatanmarz@gmail.com (Y.M.)

**Keywords:** acute kidney injury, kidney replacement therapy, internal medicine wards, hospital outcomes, healthcare system disruption

## Abstract

**Background/Objectives**: Acute kidney injury is a frequent complication among patients hospitalized in internal medicine wards and is associated with adverse outcomes. While increased AKI burden has been reported during pandemics, data on AKI during other forms of acute healthcare system disruption, including armed conflict, are limited. We evaluated AKI incidence and outcomes during a period of wartime healthcare system disruption. **Methods**: We conducted a retrospective single-center cohort study comparing internal medicine hospitalizations during the initiation of the Israel–Gaza war with a corresponding pre-war period in 2022. AKI was identified based on changes in serum creatinine during hospitalization. Clinical characteristics, AKI etiology, hospitalization indication at admission, and in-hospital outcomes were compared between periods. Multivariable regression models assessed the association between year of hospitalization and outcomes, adjusting for sex, comorbidities, admission kidney function, vital signs, and hospitalization indication. **Results**: AKI occurred in 110 of 2228 hospitalizations in 2022 (4.9%) and in 95 of 1456 hospitalizations in 2023 (6.5%), representing a 32% relative increase during the wartime period (relative risk 1.32, 95% confidence interval 1.03–1.69). Baseline demographics, comorbidity burden, admission vital signs, and kidney function were similar between cohorts. During the wartime period, hospitalizations were less frequently infection-related, and AKI etiology shifted toward a higher proportion of pre-renal and unspecified causes. Patients hospitalized in 2023 more frequently required kidney replacement therapy and were more often discharged with ongoing dialysis or to nursing facilities. After multivariable adjustment, hospitalization during 2023 remained independently associated with less favorable AKI-related outcomes. **Conclusions**: Wartime healthcare system disruption was associated with higher AKI incidence and less favorable AKI-related outcomes among internal medicine hospitalizations, independent of measured patient-level risk factors. These findings suggest that kidney outcomes may be sensitive to system-level stress during healthcare emergencies and underscore the importance of maintaining AKI prevention, monitoring, and recovery pathways under such conditions.

## 1. Introduction

Acute kidney injury (AKI) is a common and clinically significant complication among patients hospitalized in internal medicine wards, and is associated with increased morbidity, mortality, and long-term kidney dysfunction [1,2,3]. Despite advances in supportive care and increased awareness, the burden of AKI remains substantial, and outcomes are highly dependent on early recognition, timely intervention, and the broader clinical context in which care is delivered. The development of AKI is influenced not only by patient-level risk factors, such as comorbidities and acute illness severity, but also by healthcare delivery processes, including timely assessment, monitoring, and supportive management [4].

Large-scale disruptions to healthcare delivery, such as pandemics, armed conflicts, and other crises, are characterized by rapid reorganization of hospital infrastructure, reduced bed capacity, staff shortages, altered admission thresholds, and constrained diagnostic and therapeutic resources.

The COVID-19 pandemic has provided the most extensive contemporary data on AKI under healthcare system strain. Multiple studies have demonstrated increased AKI incidence, higher severity, and greater need for kidney replacement therapy (KRT) during pandemic surges compared with pre-pandemic periods, including among patients admitted for non-COVID-related conditions [5]. Importantly, these changes were observed even after accounting for demographic characteristics and comorbidities, suggesting a role for system-level factors beyond direct infectious injury [6,7].

Despite this growing body of literature, data on AKI epidemiology and outcomes during wartime or non-pandemic healthcare emergencies remain scarce. Most available evidence is derived from Intensive Care Unit (ICU) or trauma populations, with limited focus on internal medicine wards, where most hospitalized adults with AKI are managed [2,3]. Moreover, few studies have directly compared AKI characteristics and outcomes during crisis periods with matched pre-crisis intervals within the same healthcare system.

In October 2023, the outbreak of the Israel–Gaza war led to an abrupt transformation of hospital operations in southern Israel. Internal medicine wards were relocated to protected underground facilities, inpatient bed capacity was markedly reduced, and routine outpatient services were largely suspended. This abrupt transition created a natural experiment to examine the impact of acute healthcare system disruption on AKI care.

In this study, we aimed to compare the incidence, etiology, and outcomes of AKI among internal medicine hospitalizations during the first three months of the war with those observed during a corresponding pre-war period in 2022. Understanding the impact of acute healthcare system disruption on AKI incidence and AKI-related outcomes in general internal medicine wards may inform preparedness strategies and optimize kidney care delivery during future emergencies.

## 2. Materials and Methods

### 2.1. Study Design and Setting

We conducted a retrospective single-center cohort study with a historical comparison at a tertiary public hospital in southern Israel. The study compared internal medicine hospitalizations during two predefined periods: the first three months of the Israel–Gaza war in 2023 and a corresponding three-month period in 2022, which served as a reference period of routine hospital activity.

During the early war period in 2023, hospital operations were substantially modified due to emergency conditions. Internal medicine wards were relocated to protected underground facilities, inpatient bed capacity was markedly reduced, staffing was conducted under emergency protocols, and ambulatory services were largely suspended. The reduction in hospital operations was not limited to the internal medicine wards. Rather, the entire hospital underwent substantial reorganization, with total inpatient capacity reduced from approximately 650 beds under routine conditions to 290 beds during the emergency period. This reduction affected multiple departments, including both internal medicine and surgical wards. The 2022 period reflected standard inpatient care conditions.

The study was approved by the institutional ethics committee of Barzilai University Medical Center, which waived the requirement for informed consent due to the retrospective, anonymized nature of the data.

### 2.2. Study Population

All adult patients (≥18 years) hospitalized in internal medicine wards during the study periods who developed acute kidney injury (AKI) during hospitalization were included. Patients admitted for trauma-related conditions, including crush injury, were generally managed outside the internal medicine wards during the study period and were therefore not included in the present analysis, which focused exclusively on internal medicine hospitalizations. AKI cases were identified from institutional clinical databases and verified by review of laboratory results and medical records. Patients were categorized according to year of hospitalization (2022 or 2023). For epidemiological analyses, the total number of internal medicine hospitalizations during each period was used to calculate AKI incidence.

### 2.3. Definitions

AKI was defined based on changes in serum creatinine during hospitalization, consistent with contemporary KDIGO-based clinical practice [8]. Baseline creatinine was defined as the first available serum creatinine on admission. Urine output criteria were not systematically available and were therefore not used.

Kidney replacement therapy (KRT) was defined as initiation of intermittent hemodialysis or continuous kidney replacement therapy during hospitalization or continuation of dialysis at discharge.

### 2.4. Data Collection

Data were extracted from electronic medical records and included: demographics, comorbidities (diabetes mellitus, hypertension, dyslipidemia, congestive heart failure, and malignancy), kidney function at admission, maximal serum creatinine and serum creatinine at discharge, admission vital signs, hospitalization indication at admission, AKI etiology (pre-renal, intrinsic renal, post-renal, multifactorial, or unspecified) and outcomes. Laboratory testing, including serum creatinine measurements, was performed according to the clinical judgment of the treating physicians. There were no specific institutional restrictions or protocol changes regarding laboratory testing during the wartime period.

### 2.5. Outcomes

Outcomes included length of hospitalization stay (LOS), the requirement for KRT during hospitalization, in-hospital mortality, discharge with ongoing KRT and discharge disposition (home, nursing facility, transfer to another ward or hospital, or death).

### 2.6. Statistical Analysis

Continuous variables were assessed for normality using the Shapiro–Wilk test and are presented as mean ± standard deviation or median with interquartile range (IQR), as appropriate. Categorical variables are presented as counts and percentages. Comparisons between the 2022 and 2023 cohorts were performed using the Mann–Whitney U test for continuous variables and the chi-square test or Fisher’s exact test for categorical variables, as appropriate.

AKI incidence was calculated as the proportion of AKI cases among all internal medicine hospitalizations in each study period and compared using the Pearson chi-square test. Effect sizes were expressed as absolute risk difference and relative risk with 95% confidence intervals. Incidence was analyzed at the hospitalization level, consistent with the study design.

Multivariable regression analyses were performed to assess the independent association between year of hospitalization (2023 vs. 2022) and in-hospital outcomes. LOS was analyzed using negative binomial regression because LOS represents count-type data and demonstrated overdispersion. Binary outcomes, including KRT during hospitalization, in-hospital mortality, and discharge with ongoing KRT, were analyzed using multivariable logistic regression. Discharge disposition was analyzed using multinomial logistic regression, with discharge home as the reference category. Covariates included in the multivariable models were selected a priori based on clinical relevance and data availability. These included sex, major comorbidities, kidney function at admission (serum creatinine), admission vital signs, and hospitalization indication at admission. The same general set of clinically relevant covariates was applied across models in order to maintain consistency of adjustment and to reduce selective model specification.

Missing data were handled using complete-case analysis. No formal multiple imputation was performed. Because of the retrospective design and the limited number of outcome events for several endpoints, particularly dialysis-related outcomes, the adjusted models were considered exploratory and hypothesis-generating rather than definitive.

In this context, model estimates for sparse-event outcomes should be interpreted cautiously, with greater emphasis placed on the direction, consistency, and clinical plausibility of associations than on the precise magnitude of effect estimates.

Formal model assessment was primarily based on the clinical appropriateness of model selection for each outcome type and on the stability and interpretability of the resulting estimates. Nevertheless, given the sample size and event distribution, especially for less frequent outcomes, residual imprecision and limited model stability cannot be excluded. Residual confounding due to unmeasured differences in illness severity, delayed presentation, or care processes also remains possible. Results are presented as adjusted effect estimates with 95% confidence intervals, and a two-sided *p*-value <0.05 was considered statistically significant.

The authors used ChatGPT version 5.0 (OpenAI) exclusively for English language editing and improvement of manuscript readability. The AI tool was not used for data analysis, data interpretation, or scientific decision-making. All study analyses, interpretations, and manuscript content were performed and verified by the authors, who take full responsibility for the final version.

## 3. Results

### 3.1. AKI Incidence

**Figure 1:**
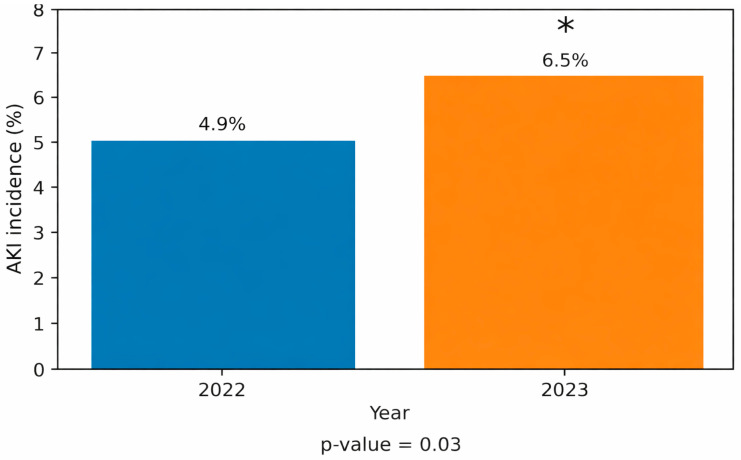
Incidence of AKI Among Internal Medicine Hospitalizations During 2023 vs. 2022. Incidence of acute kidney injury (AKI) among internal medicine hospitalizations during October–December 2023 (wartime period) compared with October–December 2022 (pre-war period). AKI incidence is presented as the proportion of AKI cases among all internal medicine admissions in each period. The relative risk (RR) of AKI during the wartime period compared with the pre-war period was 1.32 (95% confidence interval 1.03–1.69). * *p*-value < 0.03 vs. 2022.

During the study period, the total number of internal medicine hospitalizations was substantially lower compared with the pre-war period (1456 vs. 2228). Acute kidney injury (AKI) occurred in 110 of 2228 internal medicine hospitalizations in 2022 and in 95 of 1456 hospitalizations in 2023, corresponding to an incidence of 4.9% and 6.5%, respectively (Figure 1). This difference represented an absolute increase of 1.6 percentage points and a relative increase of approximately 32% in AKI incidence during the 2023 period (relative risk 1.32, 95% confidence interval 1.03–1.69).

### 3.2. Baseline Characteristics

Baseline characteristics of patients hospitalized with AKI were similar between the two study periods (Table 1). The distribution of sex and the prevalence of major chronic comorbidities, including diabetes mellitus, hypertension, dyslipidemia, congestive heart failure, and malignancy, did not differ significantly between the cohorts. Admission vital signs, including systolic and diastolic blood pressure and heart rate, were also similar between groups.

Kidney function was comparable between periods. Admission serum creatinine, maximal serum creatinine during hospitalization, and serum creatinine at discharge showed no significant differences between 2022 and 2023. In contrast, the distribution of hospitalization indications differed between periods. In 2023, a lower proportion of admissions was attributed to infectious indications, whereas a higher proportion was classified as non-specific or mixed medical indications (Table 1).

### 3.3. AKI Etiology and Hospitalization Indications

**Figure 2:**
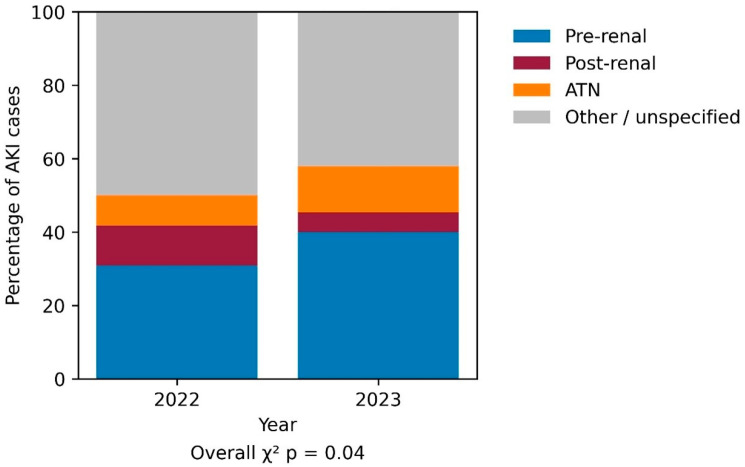
Etiology of AKI Among Internal Medicine Hospitalizations During 2023 vs. 2022. Distribution of acute kidney injury (AKI) etiologies among patients hospitalized in internal medicine wards during October–December 2023 (wartime period) compared with October–December 2022 (pre-war period). AKI etiologies were classified as pre-renal, intrinsic renal, post-renal, multifactorial, or unspecified based on clinical documentation. Overall differences in etiology distribution between periods were assessed using the chi-square test (*p*  <  0.05).

AKI etiologies were classified into the major clinically relevant categories for analysis. Less frequent specific etiologies were grouped under other/unspecified because of small numbers. The distribution of AKI etiologies differed between the two study periods (Figure 2). In 2023, a higher proportion of AKI cases were classified as pre-renal, while post-renal causes were less frequent. In addition, a larger proportion of AKI cases in 2023 were categorized as unspecified, whereas multifactorial etiologies were more commonly recorded in 2022.

**Figure 3:**
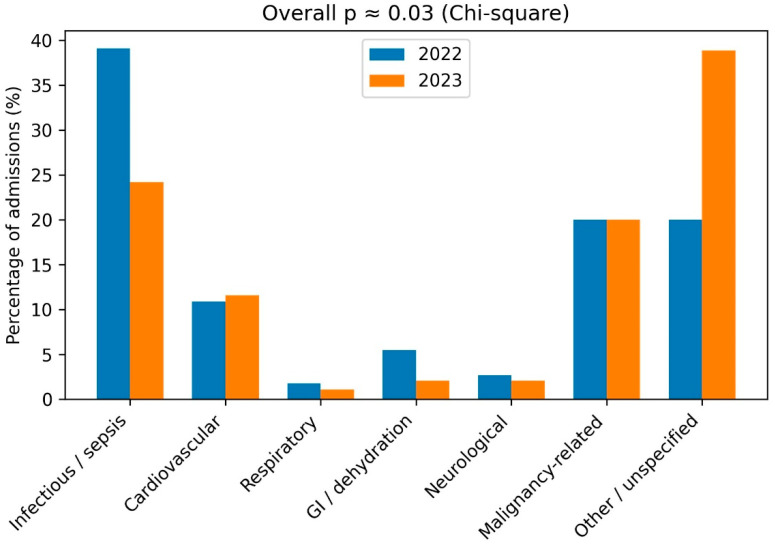
Hospitalization Indications at Admission of Patients with AKI During 2023 vs. 2022. Distribution of hospitalization indications at admission among patients with acute kidney injury (AKI) hospitalized in internal medicine wards during October–December 2023 (wartime period) compared with October–December 2022 (pre-war period). Hospitalization indications at admission were categorized as infectious/sepsis, cardiovascular, respiratory, gastrointestinal/dehydration, neurological, malignancy-related, or other/unspecified. Overall differences between periods were assessed using the chi-square test (*p*  <  0.05).

Hospitalization indications at admission also differed between the two periods (Figure 3), with a lower proportion of infection-related admissions and a higher proportion of non-specific or mixed indications during 2023 compared to 2022.

### 3.4. Outcomes

The LOS did not differ significantly between the two periods (Table 2). However, differences were observed in the clinical course of hospitalization. In 2023, kidney replacement therapy (KRT) was required more frequently during hospitalization compared with 2022. In-hospital mortality was higher during the 2023 period, although this difference did not reach conventional statistical significance in unadjusted analyses. Discharge with ongoing KRT occurred more often in 2023 than in 2022. Discharge destination also differed between the two periods (Table 2). In 2023, a lower proportion of patients were discharged home, whereas discharge to nursing facilities was more frequent. Transfers to other wards or facilities occurred at similar rates between periods.

### 3.5. Multivariable Analyses

Results of multivariable regression analyses are summarized in Table 3. After adjustment for sex, comorbidities, kidney function at admission, admission vital signs, and hospitalization indication at admission, the year 2023 remained independently associated with several in-hospital outcomes. Hospitalization during 2023 was independently associated with higher odds of requiring KRT during hospitalization and higher odds of discharge with ongoing KRT. An association between the year 2023 and increased in-hospital mortality was observed but did not reach statistical significance after multivariable adjustment. LOS was not associated with the year of hospitalization after adjustment.

In multinomial analyses of discharge disposition, hospitalization in 2023 was independently associated with a higher likelihood of discharge to a nursing facility compared with discharge home. After adjustment, no association was observed between year of hospitalization and transfer to another ward or facility, or in-hospital death. Full details of the multivariable models, including β coefficients and effect estimates for all covariates, are provided in Supplementary Table S1.

## 4. Discussion

In this retrospective single-center study, we evaluated the incidence, characteristics, and outcomes of AKI among internal medicine hospitalizations during the first three months of the 2023 Israel–Gaza war and compared them with a corresponding pre-war period in 2022. We observed a higher AKI incidence, significant shifts in AKI etiology, and less favorable AKI-related outcomes during the initial wartime period, including increased need for KRT, higher rates of discharge with ongoing KRT, and greater likelihood of discharge to nursing facilities. Notably, these associations persisted after adjustment for demographic characteristics, comorbidities, baseline kidney function, admission vital signs, and hospitalization indication at admission, suggesting that factors beyond individual patient risk may have contributed to the observed differences.

The substantially lower hospitalization volume during the wartime period likely reflects higher admission thresholds and altered triage practices, potentially enriching the hospitalized population with patients at higher clinical risk. Such case-mix enrichment could have contributed both to the higher observed AKI incidence and to the less favorable in-hospital outcomes in 2023. Accordingly, our findings should not be interpreted as proving a direct causal effect of healthcare system disruption alone, but rather as reflecting the combined impact of system-level disruption and likely differences in the clinical severity of the hospitalized population, even in the absence of marked differences in baseline comorbidity burden.

### 4.1. AKI Incidence Under Healthcare System Disruption Period

The higher incidence of AKI observed during the initial wartime period aligns with prior observations from large-scale healthcare crises, most notably during the COVID-19 pandemic. Multiple hospital-based studies reported increased AKI incidence during pandemic surges compared with preceding years, even among patients admitted for non-COVID-related conditions [5]. These findings support the concept that system-level stressors, such as altered admission thresholds, increased illness acuity, and reduced capacity for early intervention, may increase AKI risk independently of specific disease processes.

Importantly, in our cohort, baseline demographic characteristics, comorbidity burden, kidney function at admission, and admission vital signs were comparable between periods. This observation parallels reports demonstrating that increased AKI incidence during crisis periods cannot be fully explained by differences in measured patient-level risk factors alone [6,7]. The lower overall hospitalization volume during the wartime period should be considered when interpreting incidence estimates, as higher admission thresholds may have influenced case mix.

### 4.2. Shifts in Hospitalization Indications at Admission and AKI Etiology

During the initial wartime period, fewer admissions were attributed to clearly defined infectious causes, while a larger proportion were classified as non-specific or mixed medical indications. Similarly, we identified significant differences in the distribution of AKI etiologies between periods, with a higher proportion of pre-renal and unspecified AKI and fewer post-renal and multifactorial cases during the disruption period. Notably, shifts in hospitalization indications at admission paralleled changes in AKI etiology, suggesting a shared influence of system-level care processes.

Similar shifts have been described in pandemic-era cohorts, where hemodynamic instability, hypovolemia, sepsis, and limited diagnostic evaluation were more prevalent, while access to imaging and nephrology consultation was often constrained [5].

During the war period, hospital operations were profoundly altered: inpatient capacity was markedly reduced, care was delivered in protected underground facilities, patients were treated in large open spaces rather than standard ward rooms, staffing patterns followed emergency protocols, and ambulatory services were largely suspended. Such conditions may affect multiple aspects of inpatient care, including monitoring intensity, timeliness of interventions, continuity of multidisciplinary management, and discharge planning.

The increase in unspecified AKI can reflect diagnostic compression under emergency conditions rather than a true change in pathophysiology. Studies from overwhelmed healthcare systems have noted reduced frequency of diagnostic imaging, shortened clinical evaluations, and prioritization of acute stabilization over etiologic refinement, leading to broader or indeterminate AKI classifications [4,9]. Comparable challenges have been reported in trauma and conflict-related settings, where delayed presentation and limited resources complicate etiologic assessment [10,11].

### 4.3. KRT and Renal Recovery

One of the most clinically relevant findings of our study is the increased requirement for KRT during hospitalization and the higher likelihood of discharge with ongoing KRT during the disruption period. Prior studies consistently demonstrate that AKI occurring under crisis conditions is more likely to progress to severe stages requiring dialysis [12,13]. Pandemic cohorts reported acute dialysis initiation rates ranging from 2% to 9% among hospitalized patients and substantially higher rates among those with AKI, particularly during periods of ICU saturation and resource reallocation [6,7]. Moreover, incomplete renal recovery at discharge has been well documented following severe AKI requiring KRT, with significant implications for long-term kidney outcomes, healthcare utilization, and quality of life [14,15]. Our findings suggest that healthcare system disruption may exacerbate these trajectories.

Several plausible mechanisms could underlie the higher rates of KRT use and discharge with ongoing KRT observed. Delayed presentation to hospital, disruptions in outpatient follow-up prior to admission, or reduced ability to closely monitor fluid balance and hemodynamics may contribute to AKI progression. In addition, operational constraints may influence clinical decision-making thresholds for initiating KRT or prolonging renal support beyond discharge, particularly in the context of bed shortages and the need to expedite patient turnover.

### 4.4. Discharge Disposition and Functional Outcomes

Patients hospitalized during the disruption period were more frequently discharged to nursing facilities, consistent with prior studies linking AKI severity and incomplete recovery to increased need for post-acute institutional care [9,15]. This pattern highlights the broader functional and rehabilitative consequences of crisis-associated AKI, extending beyond in-hospital outcomes. This finding further suggests that functional recovery and readiness for home discharge may have been adversely affected during the emergency period.

This may reflect broader impacts of hospitalization under crisis conditions, including reduced rehabilitation resources, higher caregiver burden, or a more cautious approach to discharge planning in the setting of ongoing instability. Importantly, discharge destination has been shown to correlate with long-term morbidity and mortality in AKI survivors, underscoring the clinical relevance of this finding [3,14].

### 4.5. Mortality and Competing Risks

Although in-hospital mortality was numerically higher during the wartime period, this difference did not reach statistical significance. This finding should be interpreted cautiously. The absence of a statistically significant association with in-hospital mortality may reflect limited statistical power and the presence of competing risks, rather than a true absence of effect. Several studies have emphasized that competing risks, particularly early mortality, may obscure associations between AKI and death during crisis periods [3]. Additionally, reduced laboratory monitoring frequency and under-ascertainment of milder AKI cases may lead to detection bias, enriching identified AKI cases with more severe phenotypes [5].

### 4.6. Clinical Implications

Together, our findings support the growing recognition that AKI is not solely a patient-level phenomenon but is also sensitive to healthcare system context. Evidence from pandemics, trauma systems, and other crises consistently demonstrates that system-level disruptions are associated with higher AKI burden and worse renal outcomes [5,9,15,16]. Preserving kidney care pathways, including early identification, hemodynamic optimization, timely nephrology consultation, and post-acute planning, may therefore be critical during periods of acute system stress.

### 4.7. Limitations

Several limitations should be considered when interpreting our findings. First, this was a retrospective, single-center cohort study with a historical comparison, which may limit generalizability and does not allow causal inference. The historical comparison design is inherently vulnerable to unmeasured differences between periods, including changes in case mix, admission thresholds, hospital operations, and clinical practice. In particular, the substantially lower hospitalization volume during the wartime period may reflect stricter admission thresholds and selective hospitalization of patients with greater acute illness severity or more complex medical needs, which could have contributed to the higher AKI incidence and less favorable outcomes observed in 2023. In addition, AKI incidence was analyzed per hospitalization rather than per patient-days. Although this hospitalization-level approach was consistent with the study design and length of stay was broadly similar between periods, patient-time standardized incidence measures may provide complementary information in future studies, particularly when hospital activity and capacity differ substantially between periods.

Second, AKI was identified using serum creatinine-based criteria only, because urine output data were not systematically available in this retrospective dataset. This may have led to under-ascertainment of some AKI cases, particularly milder or transient episodes that might have been detected by urine output criteria alone. In addition, laboratory monitoring practices may have differed during the wartime period under emergency conditions, potentially affecting AKI detection and case ascertainment between periods.

Third, unmeasured confounding related to illness severity and care processes, including delayed presentation, changes in staffing ratios, nephrology consultation availability, and triage practices during the wartime period, could not be fully accounted for and may have influenced AKI recognition, management, and outcomes.

Fourth, AKI etiology and hospitalization indication were classified into major clinically relevant categories, and less frequent specific causes were grouped as other or unspecified because of small numbers; therefore, more granular etiological subclassification was not feasible. In addition, patients admitted for trauma-related conditions, including crush injury, were not managed in the internal medicine wards and were therefore not included in the present analysis.

Fifth, the limited number of events for several outcomes, particularly dialysis-related endpoints, may have reduced statistical power and resulted in imprecise estimates; accordingly, multivariable analyses should be interpreted as exploratory and hypothesis-generating. Finally, we lacked data on post-discharge kidney recovery, long-term dialysis dependence, and mortality beyond hospitalization.

Despite these limitations, the study provides a unique real-world perspective on AKI care during an extreme and sudden healthcare system disruption. By leveraging a natural experiment created by the war period and comparing it with a well-defined reference period, our findings highlight the vulnerability of patients with AKI to system-level stressors that extend beyond individual clinical risk factors.

## 5. Conclusions

In conclusion, the early phase of the war was associated with a higher incidence of AKI among internal medicine hospitalizations and with less favorable AKI-related outcomes. Although these associations persisted after adjustment for measured baseline characteristics and admission severity markers, they should be interpreted cautiously given the possibility of residual confounding, differences in case mix, and delayed presentation during the wartime period. These findings nonetheless underscore the importance of maintaining robust kidney care pathways and monitoring strategies during periods of acute healthcare system disruption.

## Figures and Tables

**Table 1:** Baseline Characteristics of Patients with AKI in 2023 vs. 2022.

Variable	2022 (*n* = 110)	2023 (*n* = 95)	*p*-Value
Demographics			
Female sex, *n* (%)	54 (49.1%)	49 (51.6%)	0.92
Comorbidities			
Diabetes mellitus, *n* (%)	55 (50.0%)	58 (61.1%)	0.93
Hypertension, *n* (%)	91 (82.7%)	73 (76.8%)	0.84
Dyslipidemia, *n* (%)	46 (41.8%)	46 (48.4%)	0.88
Congestive heart failure, *n* (%)	31 (28.2%)	24 (25.3%)	0.64
Malignancy, *n* (%)	12 (10.9%)	14 (14.7%)	0.13
Kidney Function			
Admission creatinine, mg/dL, median [IQR]	2.69 [1.77–4.11]	2.39 [1.73–3.51]	0.54
Maximal creatinine, mg/dL, median [IQR]	2.91 [2.03–4.38]	2.88 [1.85–4.15]	0.69
Discharge creatinine, mg/dL, median [IQR]	1.58 [1.20–2.33]	1.45 [0.88–2.45]	0.23

Baseline characteristics of patients with acute kidney injury (AKI) hospitalized in internal medicine wards during October–December 2022 (pre-war period) and October–December 2023 (wartime period). Data are presented as numbers (percentages) for categorical variables and medians [interquartile ranges] for continuous variables. Comparisons between groups were performed using the chi-square or Fisher’s exact test for categorical variables and the Mann–Whitney U test for continuous variables, as appropriate. A two-sided *p*-value < 0.05 was considered statistically significant.

**Table 2:** Unadjusted In-hospital Course and Outcomes of Patients with AKI in 2023 vs. 2022.

Variable	2022 (*n* = 110)	2023 (*n* = 95)	*p*-Value
Hospital course			
Length of stay, days, median [IQR]	8 [5–13]	7 [5–12]	0.40
KRT during hospitalization, *n* (%)	8 (7.3%)	14 (14.7%)	0.08
Outcomes			
In-hospital mortality (binary outcome), *n* (%)	11 (10.0%)	18 (18.9%)	0.06
Discharge with ongoing KRT, *n* (%)	4 (3.6%)	12 (12.6%)	0.015
Discharge destination			0.07
Home, *n* (%)	85 (77.3%)	63 (66.3%)	
Other ward, *n* (%)	9 (8.2%)	10 (10.5%)	
Nursing facility, *n* (%)	6 (5.5%)	15 (15.8%)	
Other hospital, *n* (%)	0 (0.0%)	0 (0.0%)	
In-hospital death (multinomial category), *n* (%)	11 (10.0%)	7 (7.4%)	

Unadjusted in-hospital course and outcomes of patients with acute kidney injury (AKI) hospitalized in internal medicine wards during October–December 2022 (pre-war period) and October–December 2023 (wartime period). Data are presented as numbers (percentages) for categorical variables and medians [interquartile ranges] for continuous variables. Between-group comparisons were performed using the chi-square or Fisher’s exact test for categorical variables and the Mann–Whitney U test for continuous variables, as appropriate. A two-sided *p*-value < 0.05 was considered statistically significant.

**Table 3:** Multivariable-Adjusted Outcomes of Patients with AKI in 2023 vs. 2022.

Outcome	Adjusted Effect Estimate	95% CI
Length of stay (days)	0.84	0.62–1.14
KRT during hospitalization	4.14	1.23–13.92
In-hospital mortality (binary outcome)	2.50	0.93–6.73
Discharge with ongoing KRT	11.89	2.23–63.30
In-hospital death (multinomial category)	0.62	0.18–2.20
Transfer/Other ward or hospital	1.2	0.38–3.79
Discharge to nursing facility	3.74	1.20–11.63

Multivariable-adjusted in-hospital outcomes of patients with acute kidney injury (AKI) hospitalized in internal medicine wards during October–December 2023 (wartime period) compared with October–December 2022 (pre-war period). Results are presented as adjusted effect estimates with 95% confidence intervals. In-hospital mortality was analyzed as a binary outcome, whereas discharge disposition was analyzed as a multinomial outcome, with in-hospital death included as one of the discharge categories. Models were adjusted for sex, comorbidities, admission kidney function, admission vital signs, and hospitalization indication at admission.

## Data Availability

The data that support the findings of this study are not publicly available due to their containing information that could compromise the privacy of research participants but are available from NLI, the corresponding author.

## Supplementary Materials

The following supporting information can be downloaded at https://www.mdpi.com/article/10.3390/jcm15082943/s1, Table S1: Full Multivariable Regression Models for In-hospital Course and Outcomes in Patients with AKI Hospitalized in Internal Medicine During 2023 vs. 2022.
