# Supplementary material for: Acute Kidney Injury in Internal Medicine Wards During a Period of Wartime Healthcare System Disruption: A Retrospective Cohort Study
Source: J Clin Med. 2026 Apr 13;15(8):2943. doi: 10.3390/jcm15082943 (PMC13116436; doi:10.3390/jcm15082943)
Supplement: Supplementary file 1 [file jcm-15-02943-s001.zip › jcm-4241130-supplementary.pdf]

	β coefficient	IRR	95% CI	p-value
**Length of Stay (days)**				
Year 2023 (vs 2022)	−0.17	0.84	0.62–1.14	0.27
Female sex	−0.05	0.95	0.72–1.26	0.74
Diabetes mellitus	0.09	1.09	0.82–1.45	0.55
Hypertension	0.04	1.04	0.73–1.48	0.82
Dyslipidemia	−0.03	0.97	0.72–1.31	0.85
Congestive heart failure	0.18	1.20	0.89–1.63	0.23
Malignancy	0.26	1.30	0.91–1.86	0.15
Admission creatinine (per mg/dL)	0.07	1.07	1.02–1.12	0.004
Admission SBP (per 10 mmHg)	−0.04	0.96	0.91–1.02	0.18
Admission DBP (per 10 mmHg)	−0.02	0.98	0.90–1.06	0.62
Admission pulse (per 10 bpm)	0.05	1.05	1.01–1.10	0.021
Hospitalization indication at admission (categorical)	—	—	—	overall <0.05
**KRT During Hospitalization**				
Year 2023 (vs 2022)	1.42	4.14	1.23–13.92	0.021
Female sex	−0.31	0.73	0.27–1.97	0.54
Diabetes mellitus	0.48	1.62	0.61–4.33	0.34
Hypertension	0.22	1.25	0.35–4.47	0.73
Dyslipidemia	−0.19	0.83	0.30–2.30	0.72
Congestive heart failure	0.66	1.94	0.69–5.45	0.21
Malignancy	0.91	2.48	0.71–8.61	0.15
Admission creatinine (per mg/dL)	0.38	1.46	1.20–1.77	<0.001
Admission SBP (per 10 mmHg)	−0.21	0.81	0.66–0.99	0.041
Admission DBP (per 10 mmHg)	−0.05	0.95	0.77–1.18	0.64
Admission pulse (per 10 bpm)	0.12	1.13	1.01–1.27	0.033
Hospitalization indication at admission (categorical)	—	—	—	overall <0.05
**In-Hospital Mortality**				
Year 2023 (vs 2022)	0.91	2.50	0.93–6.73	0.070
Female sex	−0.44	0.64	0.24–1.70	0.37
Diabetes mellitus	0.59	1.81	0.70–4.66	0.22
Hypertension	0.33	1.39	0.43–4.46	0.58
Dyslipidemia	−0.12	0.89	0.35–2.26	0.81
Congestive heart failure	0.74	2.10	0.79–5.61	0.14
Malignancy	1.08	2.94	1.01–8.55	0.048
Admission creatinine (per mg/dL)	0.29	1.34	1.10–1.63	0.004
Admission SBP (per 10 mmHg)	−0.27	0.76	0.61–0.95	0.016
Admission DBP (per 10 mmHg)	−0.06	0.94	0.76–1.17	0.58
Admission pulse (per 10 bpm)	0.14	1.15	1.02–1.30	0.022
Hospitalization indication at admission (categorical)	—	—	—	overall <0.05
**Discharge With Ongoing KRT**				
Year 2023 (vs 2022)	2.48	11.89	2.23–63.30	0.0037
Female sex	−0.28	0.76	0.20–2.83	0.68
Diabetes mellitus	0.71	2.03	0.59–6.99	0.26
Hypertension	0.41	1.51	0.30–7.55	0.61
Dyslipidemia	−0.36	0.70	0.20–2.47	0.58
Congestive heart failure	0.88	2.41	0.73–7.99	0.15
Malignancy	1.21	3.35	0.89–12.62	0.073

	β coefficient	IRR	95% CI	p-value
Admission creatinine (per mg/dL)	0.45	1.57	1.23–2.00	<0.001
Admission SBP (per 10 mmHg)	−0.19	0.83	0.65–1.05	0.12
Admission DBP (per 10 mmHg)	−0.04	0.96	0.74–1.25	0.77
Admission pulse (per 10 bpm)	0.10	1.11	0.97–1.27	0.13
Hospitalization indication at admission (categorical)	—	—	—	overall <0.05

Table S1: Full Multivariable Regression Models for In-hospital Course and Outcomes in Patient with AKI Hospitalized in Internal Medicine During 2023 vs. 2022

Supplementary Table S1. Full multivariable regression models for in-hospital course and outcomes among patients with acute kidney injury (AKI) hospitalized in internal medicine wards during October-December 2023 compared with October-December 2022. The table presents regression coefficients and effect estimates for all covariates included in each model. Length of stay was analyzed using negative binomial regression, binary outcomes using logistic regression, and discharge disposition using multinomial logistic regression. Models were adjusted for sex, comorbidities, admission kidney function, admission vital signs, and hospitalization indication at admission. A two-sided p value <0.05 was considered statistically significant.
